# Chinese medical teams in Africa: a flagship program facing formidable challenges

**DOI:** 10.7189/jogh.09.010311

**Published:** 2019-06

**Authors:** Shu Chen, Michelle Pender, Nan Jin, Michael Merson, Shenglan Tang, Stephen Gloyd

**Affiliations:** 1 Global Health Research Center, Duke Kunshan University, Kunshan, Jiangsu, China; 2 Duke Global Health Institute, Duke University, Durham, North Carolina, USA; 3 Simon-Kucher & Partners, Beijing Office, Beijing, China; 4 School of Public Health, University of Washington, Seattle, Washington, USA; * Joint first authors

In recent decades China has made the significant transformation from aid-recipient to aid-donor. China’s extensive foreign aid program includes eight categories: civil projects, goods and materials, technical cooperation, human resource development, health assistance, emergency humanitarian aid, volunteer programs and debt relief [1]. Health is a critical piece of the foreign aid program and an important avenue for China’s role in global health. Health aid is delivered through the Chinese medical teams (CMTs), hospital construction, pharmaceutical and equipment donations, and public health/health security program support including malaria control, and health professional training programs [2]. The CMT program is arguably the most prominent of these mechanisms and is often considered a key component of China’s foreign diplomacy, especially in Africa. Recently, China received high praise for the vital role it played during the 2014-2015 Ebola epidemic in West Africa. The Chinese government dispatched Chinese military medical teams to Sierra Leone and Liberia where they assisted with disease prevention and control, provided direct clinical care and health training [3]. However, the CMT program has remained mostly unchanged, in terms of its organization and management, and has undergone little internal or external evaluation since its inception more than fifty years ago. As China continues to expand south-south cooperation in the areas of economics, trade, and health, the CMTs have the potential to play an important role in improving health in Africa, particularly with the Sustainable Development Goals, and serving as a vehicle to strengthen China’s role and engagement in global health. This article synthesizes new and previous evidence on the CMTs operation, performance and impact, analyzes and discusses rising issues and challenges, and proposes ways forward.

## METHODOLOGICAL APPROACH

Sources used in this viewpoint include international and Chinese literature published over the past decade, and study reports from relevant projects and meetings/workshops on China’s health aid programs in Africa. These include findings from two CMT reports from projects carried out in 2015: one conducted by the Duke Global Health Institute with Fudan University, China, and the other by the University of Washington (UW) in collaboration with Sun Yat-Sen University, China. The Duke team conducted interviews with key stakeholders in Tanzania to assess the impact of the CMT program. Interviewees included representatives from international health donors, Tanzanian doctors working with CMTs, and a CMT member stationed in Tanzania. The UW project took place in Ghana where they interviewed health workers, university leadership, health sector donors, Ministry of Health officials, and members of the Ghana CMT. We included findings from 17 interviews with international health professionals attending the Chinese Consortium of Universities for Global Health (CCUGH) Conference in Kunshan, China in 2016, and discussions during a consultation meeting for global health professionals involved in Africa in Beijing in 2017. The focus of this meeting was on China’s Innovative Health Models in Development Assistance for Health; increasing the effectiveness of the CMT program was one of four main components discussed.

## CMT MODEL

The CMT program began in 1963, when the first CMT was sent to Algeria. Since then, China has dispatched CMTs consisting of more than 20 000 medical professionals to 51 African countries which have provided health care for over 270 million patients (as of 2017) [4,5]. In 2014 alone, 43 CMTs were working in 42 African countries [6], helping address the large gap in the African health workforce. The primary role of the CMTs is to provide expert medical services to the host population and to build capacity among health care professionals. Each province in China has been “twinned” with one or more host contry to which they send teams. CMTs usually consist of clinicians, nurses, a leader, a translator and a chef. The overall size of teams range from half a dozen to 100 people with an average of 20-30 members. African countries often make specific requests for CMT members with specialties that meet the most pressing needs of their populations. The average tenure of the teams is 1-2 years [7], however the duration of deployment has decreased over time, for as little as half a year in some cases.

**Figure 1:**
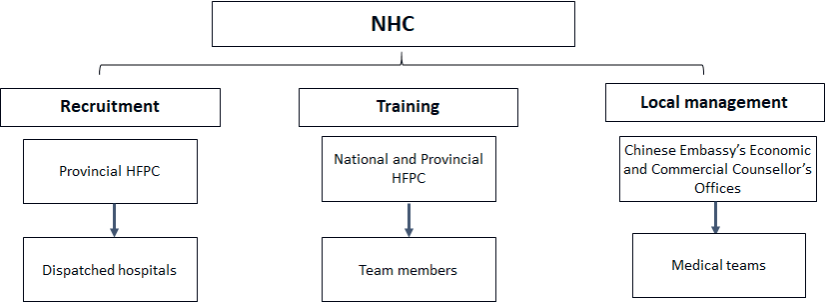
Overall organization and management structure of CMT.

The National Health Commission (NHC), the former National Health and Family Planning Commission (National HFPC), has had a strong leadership role in the overall organization and management of CMTs, including establishing recruitment parameters, training of team members and local team management (see **Figure 1**). The provincial HFPC is in charge of team member recruitment and submits their recommendations to the NHC for approval. Recruitment criteria includes: foreign language proficiency (language depends on the host country); political awareness; professional skills; health status; and maximum age of 55 [8]. Team members receive training primarily conducted by provincial HFPC on language, safety issues and diplomacy, while the NHC trains team leaders on safety, diplomacy and administrative issues. Once in the host countries, the teams are overseen by the Chinese Embassy’s Economic and Commercial Counsellor’s Office. CMTs are jointly funded through four different financial models, each with various combinations of contributions from Chinese central and local governments and the host countries.

China considers the CMTs a flagship program for its foreign aid, and lauds its significant contributions to the health of various host nations. Likewise, the CMTs are commended by many African countries for the high quality of medical care they provide, their specialist skills, reliability, and altruistic motivation to improve the health of their host populations. Tanzanian doctors interviewed for the study in Tanzania described the CMTs as “the backbone in the hospitals”, “quite skilled” and “like magic when doing surgery”. However, in several countries, Ministry of Health officials have raised concerns regarding whether the CMTs are fully utilized.

Based on Chinese government documents outlining CMT protocols throughout the years, as well as information available in the published literature, it appears that with the exception of minor adjustments to its finance and management structure, the CMT program has remained very similar since its inception in 1963. The lack of change prevents the program from growing, and reaching its full potential in the context of current health needs in recipient countries. In particular, its failure to adjust its operational system to allow recruitment to occur at a national rather than provisional level limits the pool of qualified candidates for the program. Its primary focus on direct clinical care prevents it expanding into the much needed realm of strengthening health systems. The CMT has seen a few changes such as increases in compensation (although not at a rate equal to compensation for physicians in China), and pilot programs with shorter periods of service and hospital management roles from CMTs. We explore these matters further in our findings.

## EMERGING ISSUES AND CHALLENGES

China has undergone rapid socio-economic development over the past four decades. It is now the second largest global economy and is one of the biggest donor countries and trading partners in Africa. Under such a changing context, the CMT program is increasingly facing several challenges regarding its management and administration, meeting its expected objectives, and finding its role in the health systems of low-income countries and within the international aid community.

One of the most pressing difficulties faced by CMT program management is the recruitment of qualified doctors. Our findings from the literature and interviews with CMTs in Tanzania and Ghana indicate that provinces have found it difficult to find adequately skilled health professionals willing to join the CMT team. The living standards in China, particularly those of the middle-class, such as doctors and teachers, have increased significantly, and inadequate financial incentives mean many health workers are reluctant to join the CMTs. While as mentioned earlier, the CMT program has increased its compensation for members over the years, the rates are not as high as those seen by physicians in China. Reluctance also stems from misconceived negative perceptions about Africa, long periods of absence from family, and postponing career development in the Chinese medical system. Negative feedback about the often difficult working environments, lack of equipment, drug shortages, language struggles, difficulties adjusting to different cultures, long working hours (frequently more than local medical staff), lower salaries and poorer living conditions than their colleagues from returned CMTs compound recruitment issues. As a result, some recruitment criteria are compromised, such as attaining certain professional skills, and language proficiency. Many provincial governments now recruit doctors from lower level health facilities, instead of provincial and municipal levels. This causes more challenges for the program as these individuals are less equipped to perform the duties required by the current mandate.

A common theme which emerged from interviews among Ministry of Health officials in Ghana, international health donors and local doctors in Tanzania, international health professionals attending the CCUGH conference, and the Beijing meeting was that the current operational system limits the impact of the program. The primary aim of the program has been to provide direct clinical services to local populations. While this is necessary, our study found that the CMTs could provide additional activities to build sustainable capacity of the local health services. Some teams and individuals do carry out other functions, such as training local health workers, but this is the exception rather than the rule. For the most part, the CMTs provide little in the way of structured or formal capacity building, especially outside of clinical service, which greatly limits the impact of their work. This is due to several reasons: 1) CMT program’s mandate is restricted to clinical medicine rather than broader public health issues; 2) most CMTs are not equipped or supported to expand the scope of their work; and 3) there are no resources or policies/regulations to encourage them to be innovative and creative. Furthermore, many CMTs are located in large urban hospitals that already have sufficient well-trained staff so their expertise, especially for training is less in demand.

A further constraint of the CMT program is its nature of working in isolation from the larger international aid community. Officials from Ministries of Health in Ghana and international health donors in both Ghana and Tanzania reported little to no cooperation between the CMTs and other international organizations. This limitation was confirmed by international health professional attendees at both the CCUGH conference and the Beijing meeting. While other countries such as Sweden, the Netherlands, the UK, and the US have a clear global health agenda and collaborate with other donor organizations, this is not the case with China and the CMTs. The CMT program suffers from low visibility among both Ministry of Health officials in host countries and the international donor community which limits opportunities to expand the reach of the program and to contribute to policy discussions. This represents a major lost opportunity, given China’s long experience and achievements in developing effective health policies within China.

## THE WAY FORWARD

While the CMTs have met their objective in providing high quality clinical services to populations in need, many recipient countries have gone through significant socio-economic transitions and have developed widely different needs in their health sector. To enhance China’s contribution to south-south collaboration, the SDGs, and maximize its investment in global health, especially in Africa, the old CMT mandates may no longer meet these needs. The program should be revised accordingly. Given the multiple challenges the program is now facing, we suggest several ways to improve the effectiveness and sustainability of the program and increase its role in the global health arena.

First, China should develop a cohesive health development aid program, aligning the CMT program with other health development projects. For example, China donates equipment and drugs, and constructs hospitals; linking these programs closely with the CMT programs would greatly improve the impact of both programs.

Second, subject to their qualifications and experiences, health professionals may be sent to different locations. For example, experienced highly skilled doctors may be located in specialty hospitals, while junior doctors might be more likely to thrive in rural locations, which in turn would help increase access to health care for underserved populations. While capacity building is important, the CMT program provides a necessary role in filling the gaps in direct services. A mix of clinical and preventive services is particularly useful in rural and underserved areas and would facilitate, among the CMT members, a better understanding of the entire health system. These roles might also be attractive to younger Chinese health workers who are interested in adventurous opportunities.

Third, CMT recruitment needs to be improved with appropriate financial incentives, improved living conditions, more family reunion opportunities, and more support from local health administrative systems. Recruitment could be opened up to health care workers and public health professionals who have a keen interest in improving the health of populations in low- and middle-income countries, and on their return could provide necessary global health experience to Chinese academic institutions. Open recruitment and advertisement at a national rather than provincial level might be considered. While there have been some pilot programs with shorter terms of service, the length of service in the host countries could be flexible in order to attract various categories of health professionals.

**Figure:**
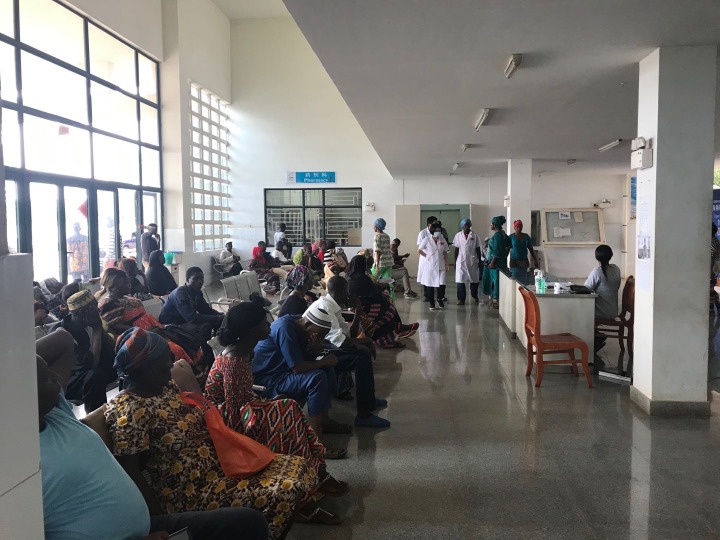
Photo: From the collection of the study team (used with permission).

Fourth, the CMT program could increase its international cooperation with other development partners. China’s health aid programs such as the CMTs should be better represented at the global level. Understanding the donor landscape in host countries, identifying common priorities, and working together with other donors would allow China to maximize the impact of its health aid and enhance China’s health diplomacy.

Fifth, China may consider adopting sector-wide approaches (SWAps), which were developed to avoid the confusion caused by fragmented donor-sponsored programs or projects. SWAps provide a cohesive way for donor countries to support national and local government policies and strategies to improve the health of people concerned. Initial evaluation of the host country’s readiness to implement programs through a broader health system approach is necessary before doing so.

Finally, China has developed a wealth of knowledge and experience as a result of its own recent health development. The CMT program should apply this knowledge in Africa. Best practices are not limited to clinical skills, but can be extended to public health practices, such as improving maternal and child health, building health information systems, and non-communicable diseases prevention and management. Traditional Chinese medicine practices can also be transferred to host countries (traditional Chinese medicine is already popular in some African countries) [9]. These potential contributions could fill an important gap in many countries.

## CONCLUSION

China has become an important donor for international health aid, and as their flagship program, the CMTs have made significant contributions to their host countries. However, the program has a few organizational and operational flaws which make recruiting well-qualified doctors difficult, and places too great an emphasis on primarily providing direct clinical service. Finally, their operational practice inhibits cooperation with the international donor community which in turn limits the impact of the program. The suggestions made in this paper are to integrate the CMT program activities with China’s other health aid programs and other international donor programs; diversify their recruitment pool; provide better financial and non-financial incentives; consider adopting a sector-wide approaches and incorporate more of China’s own experience tackling health issues in their programs. There is every reason to think that the CMT program has great potential to offer more to the improvement of health in Africa.
